# Genetic Carriers and Genomic Distribution of *cadA6*—A Novel Variant of a Cadmium Resistance Determinant Identified in *Listeria* spp.

**DOI:** 10.3390/ijms21228713

**Published:** 2020-11-18

**Authors:** Cora Chmielowska, Dorota Korsak, Barbara Szmulkowska, Alicja Krop, Kinga Lipka, Martyna Krupińska, Dariusz Bartosik

**Affiliations:** 1 Department of Bacterial Genetics, Institute of Microbiology, Faculty of Biology, University of Warsaw, Miecznikowa 1, 02-096 Warsaw, Poland; corachmiel@biol.uw.edu.pl (C.C.); b.szmulkowska@student.uw.edu.pl (B.S.); kinga.lipka@student.uw.edu.pl (K.L.); martynakrupinska1@gmail.com (M.K.); 2 Department of Molecular Microbiology, Institute of Microbiology, Faculty of Biology, University of Warsaw, Miecznikowa 1, 02-096 Warsaw, Poland; a.krop@student.uw.edu.pl

**Keywords:** *Listeria*, *Listeria ivanovii*, *Listeria seeligeri*, *Listeria monocytogenes*, heavy-metal resistance, cadmium, arsenic, plasmid, transposon, *cadA*

## Abstract

*Listeria monocytogenes* is a pathogen responsible for severe cases of food poisoning. *Listeria* spp. strains occurring in soil and water environments may serve as a reservoir of resistance determinants for pathogenic *L. monocytogenes* strains. A large collection of *Listeria* spp. strains (155) isolated from natural, agricultural, and urban areas was screened for resistance to heavy metals and metalloids, and the presence of resistance determinants and extrachromosomal replicons. Of the tested strains, 35% were resistant to cadmium and 17% to arsenic. Sequence analysis of resistance plasmids isolated from strains of *Listeria seeligeri* and *Listeria ivanovii*, and the chromosome of *L. seeligeri* strain Sr73, identified a novel variant of the *cadAC* cadmium resistance efflux system, *cadA6*, that was functional in *L. monocytogenes* cells. The *cadA6* cassette was detected in four *Listeria* species, including strains of *L. monocytogenes*, isolated from various countries and sources—environmental, food-associated, and clinical samples. This resistance cassette is harbored by four novel composite or non-composite transposons, which increases its potential for horizontal transmission. Since some *cadAC* cassettes may influence virulence and biofilm formation, it is important to monitor their presence in *Listeria* spp. strains inhabiting different environments.

## 1. Introduction

The genus *Listeria* currently comprises 20 species [1]. *Listeria* spp. are distributed ubiquitously in diverse environments including soil, surface waters, and vegetation. The presence of *Listeria* spp. in agricultural environments represents a potential source of contamination for food products [2]. *L. monocytogenes* and *L. innocua* are the species identified most frequently in foodstuffs and sites of food processing [3,4,5,6,7,8,9,10]. *L. monocytogenes* is a human and animal pathogen, so its incidence in food industries is closely monitored. The frequency with which different *Listeria* species are identified in soil and water samples varies depending on the source of isolation and geographical factors, although *L. seeligeri* was the most common isolate in a number of studies [11,12,13].

Most studies on resistance to heavy metals and metalloids in *Listeria* spp. have focused on *L. monocytogenes* strains [14,15,16,17], although a few have examined the resistance to cadmium and arsenic in other *Listeria* species [18,19,20,21]. These studies demonstrated that determinants conferring resistance to cadmium and arsenic are widely distributed among *Listeria* spp. In addition, an association between resistance to cadmium and resistance to benzalkonium chloride (BC), a sanitizer commonly used in food industries, was identified [17,19,20,22]. Notably, co-selection of cadmium and BC resistance has been observed during the conjugative transfer of plasmids between non-pathogenic *Listeria* spp. and *L. monocytogenes* [18,20].

To date, five different cadmium resistance determinants (*cadAC* efflux systems) have been identified within *Listeria* spp.: (i) the *cadA1* gene—located within transposon Tn*5422* (Tn*3* family) and commonly inserted within plasmids [23,24,25]; (ii) *cadA2*—another plasmid-associated gene [18,23,26,27]; (iii) *cadA3*—located within the integrative and conjugative element ICELm1 [28]; (iv) *cadA4*—harbored by the genomic island LGI2; and (v) *cadA5*—located within a similar island called LGI2-1 [15,29,30]. The encoded proteins CadA1–CadA3 show approximately 70% amino acid (aa) sequence identity to each other, while sharing around 35% identity with CadA4 and CadA5. The CadA4 and CadA5 proteins are more similar to one another, exhibiting around 90% identity. It is not known why cadmium resistance cassettes are so widely spread among *Listeria* spp. in the absence of strong cadmium selection pressure. The estimated worldwide mean cadmium concentration in soils is 0.36 µg/g [31], but local concentrations vary depending on the regions, soil acidity, and local pollution levels [32,33,34]. Even low concentrations of metals can induce transcription of resistance genes and potentially exert selective pressure if they result in impaired bacterial growth [30]. However, Parsons et al. [35] suggests that food processing environments may be the main source of selection pressure that leads to the acquisition of cadmium resistance determinants by *Listeria* spp. strains. Interestingly, Parsons et al. [30] and Pombinho et al. [36] observed that the presence of some *cadAC* resistance cassettes in *Listeria* can influence other phenotypic traits, such as virulence and biofilm formation, which suggests that these heavy-metal resistance determinants may have additional functions.

Arsenic resistance in *Listeria* spp. is usually chromosomally determined, either by a Tn*554*-like transposon (*arsCBADR*) or genomic islands LGI2 and LGI2-1 (*arsR1D2R2A2B1B2* and upstream *arsA1D1* cassette) [15,28]. However, the occurrence of arsenic resistance genes within *Listeria* spp. plasmids has also been observed [23,37].

The mechanisms regulating intracellular levels of copper in *L. monocytogenes* remain poorly understood. The first copper transporter identified in this species was the plasmid-encoded efflux pump CtpA from *L. monocytogenes* strain DRDC8, which, however, appears to be present in very few strains [38,39]. CtpA plays a role not only in copper uptake but also in virulence [40]. Other putative copper-translocating P-type ATPases and multi-copper oxidases have been identified via sequence analysis of *L. monocytogenes* plasmids [23], but their function has not been tested experimentally. A conserved chromosomal copper resistance operon, *csoR*-*copA*-*copZ*, was described in *L. monocytogenes* EGD [38]. This encodes the copper-sensing transcriptional regulator CsoR, a copper-exporting P1B-type ATPase CopA and the copper metallochaperone CopZ. It was also shown that the penicillin-binding protein PBP4, encoded by chromosomal gene *lmo2229*, is involved in copper tolerance in *L. monocytogenes* [41].

So far, little is known about the extrachromosomal replicons of *Listeria* spp. other than *L. monocytogenes*. The National Centre of Biotechnology Information (NCBI) database (GenBank) contains 57 complete *Listeria* spp. plasmid sequences: 52 from *L. monocytogenes*, 3 from *L. innocua*, 1 from *L. grayi*, and 1 from *L. welshimeri* (as of 30 August 2020). *Listeria* spp. plasmids can harbor genes influencing bacterial tolerance to various environmental stresses, including (i) heavy metals [23,25,27,37], (ii) sanitizers [18,20,27,42], (iii) antibiotics [43,44,45], (iv) high temperatures [46], and (v) osmotic and oxidative stress [47]. In addition, many of the plasmid-borne genes encode proteins of unknown function that may potentially contribute to bacterial survival under stressful conditions.

*Listeria* spp. strains from soil environments can potentially serve as a reservoir of resistance determinants, which, if encoded by mobile elements, may be transferred to pathogenic *L. monocytogenes* strains. The aim of this study is to characterize a collection of 155 *Listeria* spp. strains isolated from soil and water samples collected from Poland and the United States in terms of (i) their susceptibility to heavy metals and metalloids, (ii) the presence of resistance determinants, and (iii) the occurrence and distribution of plasmids commonly harboring resistance genes.

## 2. Results and Discussion

### 2.1. Isolation and Taxonomic Position of Listeria spp. Isolates

In this study, 143 *Listeria* spp. strains were obtained from 555 soil and 15 water samples were collected from diverse locations representing natural, urban, and agricultural environments. PCR analysis showed that *L. seeligeri* was the predominant species (67 strains), followed by *L. innocua* (29 strains), *L. monocytogenes* (24 strains), *L. ivanovii* (16 strains), and *L. welshimeri* (7 strains). In soil and water samples, *L. seeligeri* strains are usually identified more often than other *Listeria* species, e.g., [12,13]. A further 12 strains provided by the Food Safety Laboratory at Cornell University in the USA (3 *L. seeligeri*, 1 *L. innocua*, 4 *L. welshimeri*, 4 *L. marthii*), also originating from soil and water samples, were added to the collection. In total, 155 *Listeria* strains representing six species were examined in this study (listed in Supplementary Table S1).

### 2.2. Susceptibility of Listeria spp. Strains to Heavy Metals and Metalloids

**Figure 1:**
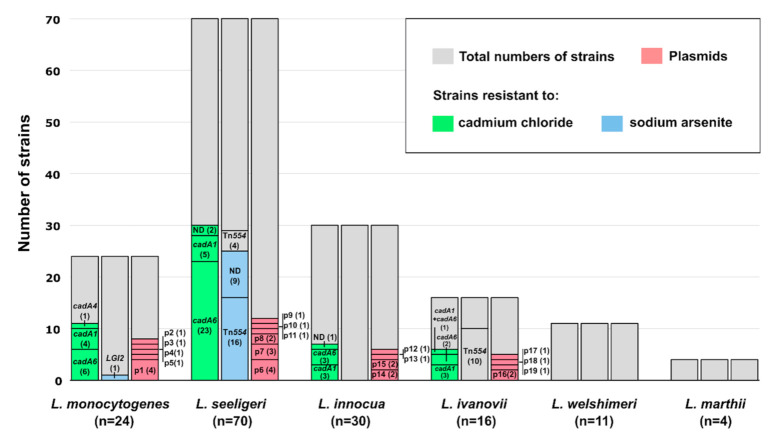
Resistance profiles, resistance determinants, and plasmids identified in *Listeria* spp. strains isolated from soil samples. Identified determinants conferring resistance to cadmium (*cadA1, cadA4, cadA6*) and arsenic (Tn*554*-associated *arsA*, LGI2-associated *arsA*); ND—undetermined resistance gene. The number of strains carrying identified resistance determinants and plasmids (representing 19 RFLP groups: p1–p19) is given in parentheses. The total number of strains within a species is indicated by the extent of the gray bar (n). Strains harboring Tn*554*-associated *arsA* genes, but not classified as resistant to arsenic, are indicated in gray.

All strains were tested for their susceptibility to heavy metals and metalloids (cadmium, arsenic, and copper) using an agar dilution assay. The presence of resistance determinants to cadmium (*cadA1*–*cadA5* genes), arsenic (*arsA1* and *arsA2* genes associated with LGI2; *arsA* gene associated with the Tn*554*-like transposon), and copper (*ctpA* gene) was determined by PCR amplification (Figure 1) using primers listed in Table 1.

The frequency of resistant strains varied depending on the species (Figure 1). Overall, 35% of the strains were determined to be resistant to cadmium and 17% to arsenic (minimum inhibitory concentrations (MICs), or lower bounds of MIC values if growth was still observed at highest tested concentrations, are presented in Supplementary Table S1). There are several reports on the prevalence of cadmium and arsenic resistance among *L. monocytogenes* strains [17,22,48] and a few studies that include other *Listeria* species [19,20,21], which indicate that resistance to cadmium is widespread, especially among food-associated isolates, while arsenic resistance is usually found at lower frequencies.

However, in contrast to *Listeria* spp. isolated from food products [16,17,18,20,21], few of the resistant strains identified in this study harbored previously known cadmium resistance determinants (Figure 1, Supplementary Table S1). We identified *cadA1* in only 16 strains (4 *L. monocytogenes*, 5 *L. seeligeri*, 4 *L. ivanovii* and 3 *L. innocua* strains) and *cadA4* in 1 strain (*L. monocytogenes*). None of the strains carried *cadA2*, *cadA3*, or *cadA5* resistance determinants. In total, only 31% of the cadmium-resistant strains harbored one of the known gene variants *cadA1–cadA5*, compared to 95% [16], 78% [18], and 61% [21] reported among food industry isolates in previous studies.

The Tn*554*-associated arsenic resistance cassette was present in most of the arsenic-resistant strains (62%). Interestingly, the Tn*554*-associated arsenic resistance cassette was also detected in 14 strains (4 of *L. seeligeri*, 10 of *L. ivanovii*) that were capable of growth at 250 µg/mL of sodium(meta) arsenite, but not at 500 ug/mL. According to the applied criteria, these strains were not considered resistant. It has yet to be determined why the presence of the resistance cassette confers tolerance only to lower concentrations of arsenic in these strains.

Cadmium- and arsenic-resistant strains that were PCR negative for the tested genes may either harbor yet to be identified resistance determinants or sequence divergence may have occurred in the DNA regions where the primers anneal.

In the case of copper compounds, there are no determined breakpoint concentrations for *Listeria* spp. Among the tested strains, only 1 *L. monocytogenes* strain (Sr118) yielded confluent growth at 2400 µg/mL copper(II) sulfate pentahydrate, while 78 strains were capable of growth at up to 1200 µg/mL. Some *Listeria* spp. plasmids harbor genes potentially involved in copper detoxification [23,39]; however, only one of them, *ctpA*, has had its role in copper tolerance experimentally confirmed [39]. The *ctpA* gene was not detected in any of the strains tested in this study.

Available data from locations close to some of the sampling areas (Warsaw city parks and Kampinoski National Park) showed relatively low median concentrations of cadmium (<1 µg/g), arsenic (<5 µg/g), and copper (<20 µg/g) in soil samples [49,50,51]. However, even low concentrations of toxic metals and metalloids can potentially exert selective pressure on bacteria.

### 2.3. Plasmid Occurrence and Distribution in Listeria spp. Strains 

Among the *Listeria* spp. strains tested in this study, 31 (20%) harbored large plasmids. The reported incidence of plasmids in other studies usually lies in the range 15–50% and is higher among food and environmental isolates than in clinical isolates [19,25,48]. The identified plasmids were classified into 19 groups (named p1–p19) based on their RFLP patterns (Supplementary Table S1). None of the tested *L. welshimeri* and *L. marthii* strains harbored plasmids. No plasmids sharing the same restriction pattern were identified in strains of different species. Interestingly, plasmids of the p1 group showed an identical RFLP pattern to those identified in 92 *L. monocytogenes* strains (isolated from food products in Poland) analyzed in our previous study [52].

### 2.4. Nucleotide Sequences of L. seeligeri and L. ivanovii Replicons Containing Resistance Determinants

**Figure 2:**
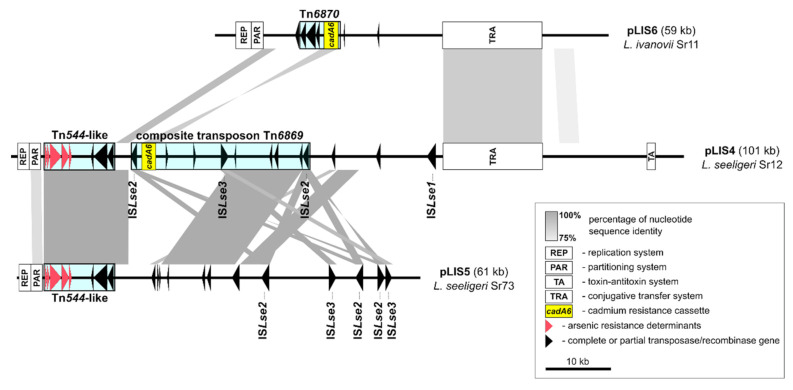
Comparative analysis of plasmids pLIS4 (*L. seeligeri* Sr12), pLIS5 (*L. seeligeri* Sr73) and pLIS6 (*L. ivanovii* Sr11). Plasmid sequences were aligned and visualized using EasyFig. Gray shading indicates conserved DNA regions with at least 75% nucleotide sequence identity of at least 1 kb in length. A simplified structure of the plasmids is presented, showing only the putative REP, PAR, TA, TRA modules, partial or complete transposase/recombinase genes and heavy-metal resistance determinants.

*Listeria* spp. plasmids often carry cadmium resistance determinants and can contribute to the horizontal transfer of this resistance phenotype [18,20,25]. All strains harboring plasmids of the p6 (*L. seeligeri*, 4 strains) and p8 (*L. seeligeri*, 2 strains) RFLP profiles were resistant to cadmium, while not containing any of the known *cadA1*–*cadA5* cadmium-resistance determinants. Only one of the two cadmium-resistant *L. ivanovii* strains harboring a p16 profile plasmid encoded a known resistance determinant (*cadA1*). Therefore, the complete nucleotide sequences of the plasmids from the following strains were determined: *L. seeliegri* Sr12 (named pLIS4; plasmid profile p6), *L. seeligeri* Sr73 (pLIS5; p8), and *L. ivanovii* Sr11 (pLIS6; p16). Two of these plasmids, pLIS4 and pLIS6, contained a new variant of the *cadAC* efflux system, named *cadA6* (Figure 2). Plasmid pLIS5 did not encode any cadmium resistance determinant, however the *cadA6* resistance cassette was identified in the complete genome sequence of its host strain (*L. seeligeri* Sr73), determined in this study. Comparative analysis of the chromosomes of Sr73 (2,867,282 bp) and *L. seeligeri* SLCC3954 (the only available complete genomic sequence of this species; NC_013891.1) is presented in Supplementary Figure S2.

These are the first published complete sequences of plasmids from *L. seeligeri* and *L. ivanovii* species. The largest of these plasmids, pLIS4, is 101,192 bp in length and contains 102 predicted protein-coding sequences (CDSs), including 14 pseudogenes. pLIS5 is 60,659-bp long and encodes 77 CDSs (15 pseudogenes), while pLIS6 is 59,142-bp long with 68 CDSs (3 pseudogenes) (Figure 2). The plasmids share few regions of nucleotide sequence similarity with other complete plasmid sequences available in the NCBI GenBank database. The shared regions are mostly limited to transposable elements (TEs). In contrast, many *L. monocytogenes* plasmids from food-associated isolates share similar genetic backbones and show a high level of sequence conservation [23,37,53,54]. The CDSs identified in these plasmids are described in Supplementary Table S2.

The positions of the predicted genetic modules responsible for replication (REP), stabilization (PAR) and conjugative transfer (TRA) are shown in Figure 2. Large *Listeria* spp. plasmids are usually categorized into two major groups based on replication initiation protein (RepA) phylogeny [23]. However, the RepA proteins of plasmids pLIS4–6 are placed in two additional and distinct phylogenetic groups (Supplementary Figure S3). The replication proteins of plasmids pLIS5 and pLIS6 cluster together with the RepA of plasmid pLMIV from *L. monocytogenes* strain FSL J1-208 (GenBank acc. no. NZ_CM001470.1) [55], while the pLIS4 RepA is located on a separate branch. The putative PAR modules encode two proteins probably involved in active partitioning of plasmid molecules during cell division and are present adjacent to the *repA* gene in all three analyzed plasmids (Figure 2).

In addition, we identified a 15-kb-long sequence representing a possible conjugative transfer module (TRA) that is shared by pLIS4 and pLIS6 (85% nucleotide sequence identity), but absent from pLIS5 (Figure 2). This module is also present in 2 *L. seeligeri* sequence contigs (probable plasmid sequences) present in the NCBI database (NZ_JAASVW010000014.1; NZ_JAASVC010000008.1). This TRA module includes genes encoding two putative type IV secretion system proteins, a relaxase of the MobL family (protein required for initiation of plasmid conjugal transfer), a lysozyme family protein, and nine hypothetical proteins of unknown function. However, no transconjugants containing pLIS4 or pLIS6 were obtained in a bi-parental mating with a streptomycin-resistant *L. monocytogenes* strain 10403S. The strain *L. welshimeri* 40/70 [18] harboring a conjugative plasmid pLIS1 was used in a control mating, and this plasmid was transferred with high frequency. Therefore, plasmids pLIS4 and pLIS6 are either non-self-transmissible or require specific conditions for efficient transfer.

The three sequenced plasmids also contain numerous predicted TEs and genes encoding recombinases and integrases, both complete and partial, which represent traces of previous insertion events. All of the identified insertion sequences (ISs), including three novel ones, IS*Lse1*, IS*Lse2*, IS*Lse3* (the first defined ISs of *L. seeligeri*; ISFinder database), were classified as members of the IS*3* family. In addition, plasmids pLIS4 and pLIS5 harbor a Tn*554*-like arsenic resistance transposon. This transposon is also present in some *L. monocytogenes* plasmids [37], although it is usually chromosomally encoded [15,28]. Furthermore, two novel putative transposons carrying the *cadA6* cadmium-resistance cassette were identified in plasmids pLIS4 and pLIS6 (Figure 2).

### 2.5. Identification of cadA6—A Novel Cadmium Resistance Cassette Variant

**Figure 3:**
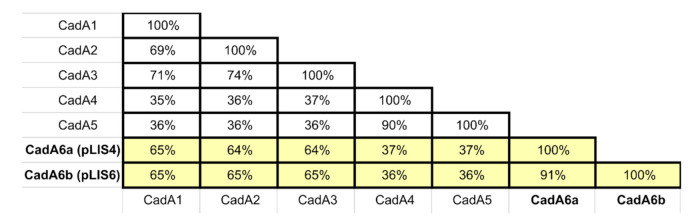
Percentage amino acid sequence identity (pairwise) of different CadA cadmium resistance determinant variants: CadA1 (GenBank acc. no. WP_003728466.1), CadA2 (WP_003726381), CadA3 (WP_010989665.1), CadA4 (WP_003744464.1), CadA5 (WP_047584121.1), CadA6a (plasmid pLIS4) and CadA6b (plasmid pLIS6).

Pairwise comparisons revealed that the novel CadA6 protein shares approximately 65% aa sequence identity with CadA1–CadA3, and 36% with CadA4–CadA5 (Figure 3 and Supplementary Figure S4). The *cadA6* sequences identified in plasmid pLIS4 and the *L. seeligeri* Sr73 chromosome are identical (named *cadA6a*), but the determinant present in pLIS6 is slightly divergent (91% aa and 86% nucleotide sequence identity), so was named *cadA6b.* A primer pair, cadA6-F/cadA6-R, was designed to enable detection of both *cadA6a* and *cadA6b* in *Listeria* spp. strains (Table 1, Supplementary Figure S5). These primers yielded strong PCR products for positive control strains (*L. seeligeri* Sr12, *L. ivanovii* Sr11), while no products of the expected size were produced with control strains containing *cadA1, cadA2, cadA3,* or *cadA4* genes. PCR analyses revealed that 65% of the cadmium-resistant strains tested in this study harbored a *cadA6* efflux system (Supplementary Table S1). Therefore, *cadA6* was the most prevalent cadmium resistance determinant identified in this study, being present in four species: *L. monocytogenes, L. seeligeri, L. innocua*, and *L. ivanovii*. The PCR products from one representative strain of each species (*L. monocytogenes* Sr119, *L. seeligeri* Sr12, *L. innocua* Sr103, *L. ivanovii* Sr11) were sequenced to confirm the specificity of the amplification reaction. Comparative analysis with the GenBank database showed that CDSs homologous to CadA6 (91–100% aa sequence identity, 100% query coverage) are present in species of various Gram-positive genera, including *Listeria*, *Bacillus*, *Solibacillus*, and *Enterococcus*.

### 2.6. Transposons Harboring the cadA6 Efflux System

**Figure 4:**
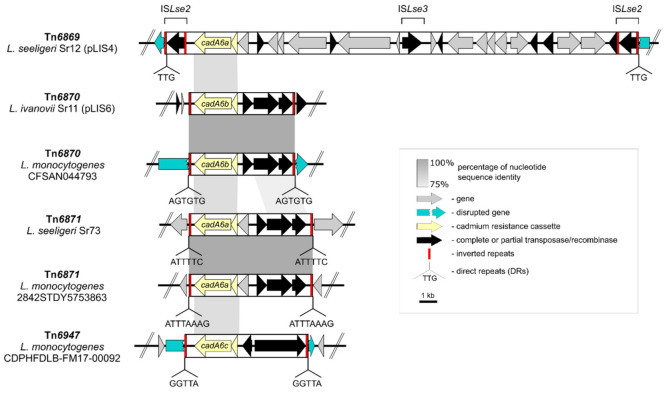
Comparative analysis of transposons Tn*6869* (*L. seeligeri* Sr12), Tn*6870* (*L. ivanovii* Sr11), Tn*6871* (*L. seeligeri* Sr73), and Tn*6947* (*L. monocytogenes* CDPHFDLB-FM17-00092), harboring the *cadA6* cadmium resistance cassette. Sequences were aligned and visualized using EasyFig. Tn*6870* present in *L. monocytogenes* CFSAN044793 (GenBank acc. no. NZ_NYDG01000002) and Tn*6871* from *L. monocytogenes* 2842STDY5753863 (NZ_FFER01000001.1) were included in the analysis. Gray shading indicates DNA regions with at least 75% nucleotide sequence identity of at least 100 bp in length. The sequences of direct repeats flanking the transposons are shown beneath each diagram. The inverted repeat sequences are not identical (no. of identical base pairs/total length of IR): Tn*6869* (22/30), Tn*6870* (39/48), Tn*6871* (41/47), Tn*6947* (35/38).

The *cadA6* cadmium resistance determinants in plasmids pLIS4, pLIS6 and the chromosome of *L. seeligeri* Sr73 are localized within three novel putative TEs (Figure 4). The *cadA6a* cassette present in pLIS4 is located within a 27-kb-long composite transposon, named Tn*6869*, formed by two flanking copies of IS*Lse2* (IS*3* family). This putative mobile element disrupts a hypothetical gene and is flanked by 3-bp-long direct repeats (DRs), which are probably the result of target site duplication upon transposition. Other copies of IS*Lse2* found in *Listeria* sequences present in GenBank also feature 3-bp-long DRs.

In pLIS6, the *cadA6b* cassette was probably introduced on a 6-kb-long non-composite transposon. Apart from the *cadA6b* efflux system, this novel transposon Tn*6870* encodes a transposase, a serine recombinase, and an ATPase, which shows aa sequence similarity to the CDSs of transposon Tn*552* (6.5 kb) from *Staphylococcus aureus* (GenBank acc. no. X52734.1). No DRs flanking Tn*6870* were identified in pLIS6. However, we discovered the same putative transposon in the genome sequences of several *L. monocytogenes* strains in the GenBank database (e.g., NZ_NYDG01000002), where it is flanked by 6-bp-long DRs. These *L. monocytogenes* strains originated from Italy and are food-associated or clinical isolates.

Another non-composite *cadA6a*-harboring transposon, Tn*6871* (7 kb), was identified in the *L. seeligeri* Sr73 chromosome sequence (nucleotide positions of TEs identified in the chromosome are presented in Supplementary Table S2). This putative transposon has a similar structure to Tn*6870*, but harbors an additional methyltransferase domain-containing protein. Identification of an empty insertion site in the chromosome of a related *L. seeligeri* strain RR4 (CP034772) enabled definition of the transposon borders with flanking 6-bp-long DRs. Tn*6871* is present in the genomic sequences (99–100% nucleotide sequence identity) of several strains of *L. monocytogenes* (e.g., NZ_FFER01000001), *L. ivanovii* (e.g., KR780025), *L. seeligeri* (e.g., NZ_JAATNZ010000010.1), and *L. innocua* (e.g., NZ_JRYX01000002). In many of these cases the transposon is flanked by 7- or 8-bp-long DRs of the target site. This transposon was found in both food-associated and clinical isolates originating from various locations, including (i) China (NZ_VOXH01000052; source—delicatessen), (ii) Switzerland (NZ_QYIR01000006; raw sausage), (iii) Ireland (NZ_LABG01000002; milk), (iv) Italy (NZ_NXUN01000005; smoked salmon), (v) Netherlands (NZ_FFGL01000001, NZ_FFER01000001; blood, cerebrospinal fluid), and (vi) Canada (NZ_QADA01000002; food).

In addition, a Tn*3* family transposon, Tn*6947* (7 kb), harboring a *cadA6* cassette was identified in a few *L. monocytogenes* strains originating from the United States (e.g., strain CDPHFDLB-FM17-00092, NZ_PQHI01000019), for which draft sequences are available in the GenBank database. The putative transposon disrupts a DNA polymerase gene and is flanked by 5-bp-long DRs. It encodes a Tn*3*-family transposase, a resolvase and a slightly divergent *cadA6* cassette (named *cadA6c*). CadA6c shows around 91% aa sequence identity to CadA6a (86% nucleotide sequence identity). The transposase shows around 43% aa sequence identity to that of Tn*5422*.

In all the analyzed cases, *cadA6* is harbored by putative transposons, which increases the potential for its dissemination. Two other cadmium resistance determinants frequently encountered in *Listeria* spp., *cadA1* and *cadA2*, are also carried by TEs–Tn*5422* a Tn*3*-family transposon [24] and a putative composite transposon formed by flanking IS*3*-family ISs [27], respectively.

### 2.7. Functional Analysis of the cadA6 Resistance Cassette

To test whether *cadA6* encodes a functional efflux system conferring cadmium tolerance, plasmid pLIS4 was removed from *L. seeligeri* Sr12 cells by repeated subculturing in BHI broth supplemented with subinhibitory concentrations of novobiocin. Plasmid-cured strains (Sr12-C1, Sr12-C2) were indeed incapable of growth on BHI agar plates supplemented with cadmium chloride (35 µg/mL). Despite repeated attempts, we did not succeed in obtaining a derivative of *L. ivanovii* Sr11 that had been cured of plasmid pLIS6. Interestingly, plasmid pLIS4 encodes a putative toxin-antitoxin (TA) stabilization system, while pLIS6 from *L. ivanovii* Sr11 does not contain such addiction modules.

Next, the putative cadmium resistance cassettes *cadA6a* (pLIS4, *L. seeligeri* Sr12) and *cadA6b* (pLIS6, *L. ivanovii* Sr11) were amplified by PCR and cloned into the mobilizable *E. coli*-*Listeria* spp. shuttle vector pDKEL. The constructed plasmids (pDKEL_cadA6a and pDKEL_cadA6b) were transferred from *E. coli* into a cadmium-susceptible *L. monocytogenes* strain 10403S. The obtained transconjugants acquired cadmium tolerance and were able to grow at a cadmium chloride concentration of 75 µg/mL. In contrast, both the recipient strain *L. monocytogenes* 10403S and a 10403S derivative carrying “empty” vector pDKEL were incapable of growth at cadmium chloride concentrations of ≥20 µg/mL. The cloned *cadA6a* and *cadA6b* efflux systems did not seem to influence the susceptibility of *L. monocytogenes* 10403S to copper(II) sulfate pentahydrate or zinc chloride, which is consistent with previous reports [24,30], that the *cadA1–cadA4* cassettes did not confer resistance to zinc. Our results strongly support the predicted cadmium detoxification function of the *cadA6* efflux system.

## 3. Materials and Methods

### 3.1. Bacterial Strains and Growth Conditions

A total of 143 *Listeria* spp. strains (listed in Supplementary Table S1) were isolated from soil and water samples collected in this study (see Section 3.2), while another 12 were obtained from the Food Safety Laboratory collection at Cornell University (USA) (*L. seeligeri*: FSL S4-0003, FSL S4-0057, FSL S4-0073; *L. innocua*: FSL H4-0088; *L. welshimeri*: FSL H4-0277, FSL S4-0070, FSL S4-0101, FSL S4-0105; *L. marthii:* FSL S4-0120, FSL S4-0696, FSL S4-0710, and FSL S4-0965). *Escherichia coli* DH5α pir [56] and streptomycin-resistant *L. monocytogenes* strain 10403S (serotype 1/2a) [57] were used to clone and analyze the *cadA6* cadmium-resistance determinant. Unless otherwise specified, bacteria were grown in brain–heart infusion (BHI) broth (BioMaxima, Lublin, Poland) or on BHI agar plates (1.5% *w/v* agar) at 37 °C. When necessary, the media were supplemented with kanamycin (Km; BioShop, Burlington, Canada)—50 μg/mL, streptomycin (Str; BioShop)—100 μg/mL for *L. monocytogenes* 10403S, erythromycin (Erm; Sigma-Aldrich, Saint-Louis, MO, USA)—2 μg/mL for *Listeria* spp., and 250 μg/mL for *E. coli.* For long-term storage, the isolates were suspended in BHI containing 20% glycerol and frozen at −80 °C.

### 3.2. Soil and Water Sample Collection and Isolation of Listeria spp.

For this study, 555 soil and 15 water samples were collected from 53 different locations in Poland in the years 2016 and 2017. Samples were collected from natural environments (328 samples), and urban (171) and agricultural (71) areas. *Listeria* spp. were isolated from these samples using a modified PN-EN ISO 11290-1 procedure. Briefly, 10 g of a soil sample or 10 mL of a water sample was added to 90 mL of selective enrichment half-Fraser broth (BioMaxima) and incubated at 30 °C for 24 h. A sample of 100 µL of the enriched culture solution was then added to 10 mL of Fraser Broth (BioMaxima) and incubated at 37 °C for 48 h. After incubation, 10 µL of the culture solution was streaked onto Chromogenic Listeria Lab-Agar plates (BioMaxima) and incubated at 37 °C for 48 h. Blue-green colonies were re-streaked onto Chromogenic Listeria Lab-Agar and BHI plates. If a single sample gave colonies displaying a distinctly different morphology on Chromogenic Listeria Lab-Agar (e.g., a different shade of color, with or without an opaque halo), representatives of each colony type were picked for further analysis.

### 3.3. Identification of Listeria Species

*Listeria* species were differentiated based on the multiplex PCR analysis, as described previously [58]. The following control strains were used: *L. monocytogenes* ATCC 13932, *L. grayi* ATCC 25401, *L. welshimeri* ATCC 35987, *L. seeligeri* ATCC 35967, *L. innocua* PZH 5/04, and *L. ivanovii* PZH 7/04 from the collection of the Department of Molecular Microbiology, University of Warsaw (Poland). In the case of presumptive *Listeria* spp. strains for which the species could not be determined by multiplex PCR, 16S rDNA amplicons (primers 27f/1492R; [59]) were sequenced.

### 3.4. Bacterial Mating between Listeria spp. Strains

Bi-parental matings between *Listeria* spp. strains were performed on solid BHI medium using *L. seeligeri* Sr12, *L. ivanovii* Sr 11, and *L. welshimeri* 40/07 [18] as donor strains, and streptomycin-resistant *L. monocytogenes* 10403S as the recipient. The mating experiments were performed in four independent repetitions at two different temperatures. Overnight cultures of the donor and recipient strains were mixed at a ratio of 1:2 and 100 μL samples of these mixtures were spread on solidified BHI medium and incubated at 30 or 37 °C for 24 h. Bacteria were then washed off the plates with BHI medium and suitable dilutions were plated on selective media containing streptomycin (100 μg/mL; selective marker of the recipient strain) and cadmium chloride (25 μg/mL), and incubated at 30 or 37 °C for up to 96 h. Transconjugants were verified by PCR using *L. monocytogenes*-specific primers and by plasmid DNA isolation.

### 3.5. Heavy-Metal and Metalloid Susceptibility

The susceptibility of *Listeria* spp. to heavy metals was determined using an agar dilution method as described previously [19], with some modifications. Briefly, several colonies picked from BHI plates were suspended in saline solution to a turbidity of 0.5 McFarland units. Spots of 3 µL of each bacterial suspension were applied to cation-adjusted Mueller Hinton 1.2% agar plates (Becton, Dickinson and Company, Frankling Lakes, NJ, USA), supplemented with 2.5% defibrinated horse blood and 0, 35, 75, 150 μg/mL (which corresponds to: 0, 0.19, 0.41, 0.82 mM) cadmium chloride (Sigma-Aldrich) or 0, 250, 500, 1000 μg/mL (0, 1.92, 3.85, 7.70 mM) sodium (meta)arsenite (Sigma-Aldrich), or to BHI plates supplemented with 0, 600, 1200, 2400 μg/mL (0, 2.40, 4.81, 9.61 mM) copper(II) sulfate pentahydrate. The plates were then incubated at 37 °C for 48 h (cadmium chloride, sodium (meta) arsenite) or 72 h (copper(II) sulfate pentahydrate). Strains were classified as resistant to heavy metals if they produced confluent growth on plates containing ≥75 μg/mL (0.41 mM) cadmium chloride or ≥500 μg/mL (3.85 mM) sodium arsenite. In the case of copper compounds, there are no determined breakpoint values for *Listeria* spp.

### 3.6. Isolation of Genomic DNA 

Genomic DNA was extracted from bacterial cells using a Chelex-100 resin-based technique. Colonies were suspended in 50 μL of 5% Chelex-100 (Bio-Rad, Hercules, CA, USA), incubated at 95 °C for 20 min, then cooled on ice for 5 min and centrifuged at 2400× *g* for 3 min. The DNA-containing supernatants were then used as the templates for PCR amplifications.

### 3.7. Plasmid DNA Isolation and Restriction Fragment Length Polymorphism (RFLP) Profile Analysis

Each strain was cultured overnight in BHI broth at 37 °C. Samples of 3 mL were centrifuged and the cell pellets resuspended in 500 μL of diluted SSC buffer (15 mM sodium chloride, 1.5 mM trisodium citrate, pH 7.0). The cells were collected by centrifugation and resuspended in 500 μL of SET buffer (25% sucrose (*w*/*v*), 50 mM EDTA, 50 mM Tris, pH 8.0, 5 mg/mL lysozyme). After incubation at 37 °C for 30 min, plasmid DNA was isolated according to the alkaline lysis procedure [60]. Purified plasmid DNA was digested with EcoRI according to the manufacturer’s protocol (Thermo Scientific in the case of all restriction enzymes used in this study). The resulting DNA fragments were separated by electrophoresis in 0.8% agarose gels. Plasmid DNA for sequencing was isolated using a larger scale preparation method [61]. Sequence assembly was verified by RFLP analysis (enzymes: EcoRI, BamHI and NcoI).

### 3.8. Plasmid Curing

Plasmid pLIS4 was removed from cells of *L. seeligeri* Sr12 as described previously [62], with some modifications. The strain was grown overnight at 37 °C in 2 mL of BHI broth supplemented with a subinhibitory concentration (0.15 µg/mL) of novobiocin (Sigma). The resulting culture was subcultured (1:100) in the same medium and again grown overnight at 37 °C. This procedure was repeated each day for 15 days. Dilutions of the final culture were then plated on Chromogenic Listeria Lab-Agar plates, and incubated at 37 °C overnight. Single colonies were screened for the presence of plasmids by PCR using primers targeting the *cadA6* gene. Negative and positive controls were used in all PCR experiments. Plasmid loss was confirmed by plasmid DNA extraction and by testing the ability of strains to grow on BHI plates supplemented with 35 and 75 μg/mL cadmium chloride.

### 3.9. Detection of Resistance Determinants by PCR

The presence of resistance determinants was detected by PCR performed with DreamTaq polymerase (Thermo Scientific, Waltham, MA, USA) using primers listed in Table 1. A primer pair for the novel *cadA6* gene was designed based on the sequences of plasmids pLIS4 (*L. seeligeri* Sr12) and pLIS6 (*L. ivanovii* Sr11). The following thermocycle conditions were applied: initial denaturation for 5 min at 95 °C; 35 cycles of denaturation at 95 °C for 30 s, annealing for 30 s at the temperatures listed in Table 1, and elongation at 72 °C for 45–150 s, depending on the expected product size; final elongation at 72 °C for 5 min. Positive control strains were included for all tested genes, apart from *cadA5* and *ctpA* for which no such controls were available. The following control strains were used: (i) *L. innocua* 62/06 [18]—*cadA1* gene, (ii) *L. welshimeri* 49/06 [18]—*cadA2* gene, (iii) *L. monocytogenes* EGDe [63]—*cadA3* gene, (iv) *L. monocytogenes* Lmo28 [52]—*cadA4, arsA1*, and *arsA2* genes associated with LGI2, and (v) *L. monocytogenes* 2270/03 [52]—*arsA* gene associated with the Tn*554-*like transposon. The PCR products amplified from positive control strains were sequenced.

### 3.10. Functional Analysis of the cadA6 Resistance Cassette

#### 3.10.1. Construction of a Mobilizable Shuttle Vector pDKEL

Plasmid pDKEL was constructed based on mobilizable *E. coli* vector pDS132 [64]. The construction of pDKEL and its derivatives is described in detail in Supplementary Figure S1. Briefly, a kanamycin resistance cassette, obtained from pDIY-Km [65] was cloned in plasmid pDS132. Then, a 3707-bp restriction fragment from this construct was ligated with an erythromycin resistance cassette obtained from plasmid pHP13 [66]. Next, the *lacZ* multiple cloning site (MCS) from plasmid pBBR1MCS was added to the construct [67]. Finally, a replication system, functional in Gram-positive bacteria, obtained from plasmid pHP13, was cloned into the MCS. The resulting plasmid pDKEL is a mobilizable *E. coli—Listeria* spp. shuttle vector containing kanamycin (selection marker for *E.coli* strains) and erythromycin (selection marker for *L. monocytogenes* strains) resistance cassettes.

#### 3.10.2. Cloning of the Cadmium Resistance Cassette into pDKEL

Novel cadmium resistance cassettes (genes *cadA6* and *cadC6*) were amplified from plasmids pLIS4 (*cadA6*a; *L. seeligeri* strain Sr12; nucleotide position: 18,685–21,508) and pLIS6 (*cadA6*b; *L. ivanovii* strain Sr11 nucleotide position: 12,664–15,715) using Phusion High-Fidelity DNA Polymerase (Thermo Scientific) and the primer pairs cadA6aF/cadA6aR and cadA6bF/cadA6bR (Table 1). The amplified DNA fragments (2843- and 3072-bp-long, respectively) and pDKEL were digested using enzymes SpeI and NotI, ligated using T4 DNA ligase, and the mixtures used to transform chemically competent *E. coli* DH5α pir with selection on LA plates containing kanamycin. Plasmid DNA was isolated from transformants and restriction analysis was used to confirm their identity as recombinants.

#### 3.10.3. Introduction of Constructed Plasmids into *L. monocytogenes* 10403S

The plasmids pDKEL_cadA6a, pDKEL_cadA6b, and empty pDKEL were introduced into the cadmium-sensitive and streptomycin-resistant *L. monocytogenes* strain 10403S by conjugation. Overnight cultures of the donor (*E. coli* DH5α pir strains harboring pDKEL_cadA6a, pDKEL_cadA6b or pDKEL), recipient (*L. monocytogenes* 10403S), and helper (*E.coli* DH5α harboring helper plasmid pKR2013; [68]) strains were harvested by centrifugation and the cells were washed to remove the antibiotics. The cell suspensions were mixed in a 1:1:1 ratio and 100 μL of the mixtures were spread on BHI agar plates. After incubation at 37 °C for 24 h, bacterial cells were washed off the plates with BHI medium and dilutions were plated on BHI agar plates supplemented with streptomycin, and depending on the introduced vector, erythromycin (in the case of pDKEL) or 20 μg/mL of cadmium chloride (pDKEL_cadA6a and pDKEL_cadA6b). These plates were then incubated at 37 °C for up to 48 h to allow transconjugant colonies to form. The presence of transferred plasmids in these strains was confirmed by restriction analysis of isolated plasmid DNA.

#### 3.10.4. Heavy-Metal Susceptibility of *L. monocytogenes* 10403S Strains Harboring the Constructed Plasmids

The susceptibility of the following strains to cadmium was tested using the agar dilution method: (i) *L. monocytogenes* 10403S carrying pDKEL_cadA6a, (ii) *L. monocytogenes* 10403S carrying pDKEL_cadA6b, (iii) *L. monocytogenes* 10403S carrying empty pDKEL, (iv) *L. monocytogenes* 10403S without any plasmids (negative control), (v) *L. seeligeri* Sr12 harboring the *cadA6*a cassette, and (vi) *L. ivanovii* Sr11 harboring the *cadA6*b cassette (positive controls). Assay plates were supplemented with cadmium chloride at 0, 35, 75, 150, and 300 μg/mL. The experiment was performed in two independent repetitions. The same strains were used in agar dilution assays to test their susceptibility to copper(II) sulfate pentahydrate (0, 600, 1200, 2400 μg/mL) and zinc chloride (0, 500, 1000, 2000, 3000 μg/mL), in order to determine whether the *cadA6* efflux system influences the susceptibility to these metals. 

### 3.11. DNA Sequencing

Sequencing of plasmids pLIS4 (*L. seeligeri* Sr12), pLIS5 (*L. seeligeri* Sr73), and pLIS6 (*L. ivanovii* Sr11), as well as the *L. seeligeri* Sr73 chromosome was performed in the DNA Sequencing and Oligonucleotide Synthesis Laboratory (oligo.pl) at the Institute of Biochemistry and Biophysics, Polish Academy of Sciences. The sequences were obtained using a combination of MiSeq (Illumina, San Diego, CA, USA) and GridION (Oxford Nanopore Technologies, Oxford, UK) sequencing and assembled with Unicycler [69]. The nucleotide sequences of the plasmids have been deposited in GenBank (NCBI, Bethesda, MD, USA) under the accession numbers MW124301 (pLIS4), CP063072 (pLIS5), MW124302 (pLIS6), and CP063071 (chromosome of *L. seeligeri* Sr73).

### 3.12. Bioinformatic Analysis

Automatic annotation of the nucleotide sequences was performed using RAST on the PATRIC platform [70], followed by manual refinement in Artemis [71], based on the homology searches (BLAST) of the NCBI database (http://www.ncbi.nlm.nih.gov). Sequences were aligned and visualized using EasyFig [72]. Insertion sequences were identified using the ISfinder website and the nucleotide sequences of novel elements were deposited in the ISfinder database [73]. Transposon numbers were registered at the Transposon Registry [74]. The phylogenetic tree of the RepA proteins of complete *Listeria* spp. plasmid sequences available in the GenBank database and plasmid pOX2 of *Bacillus anthracis* was constructed in MEGAX [75] using the maximum-likelihood algorithm (Le and Gascuel model). Statistical support for the internal nodes was determined by 1000 bootstrap replicates. Multiple sequence alignments were performed using MUSCLE [76] and visualized using Jalview [77].

## 4. Conclusions

Analysis of the first complete sequences of plasmids from *L. seeligeri* and *L. ivanovii* as well as the chromosome of *L. seeligeri* Sr73 identified a novel variant of the *cadAC* efflux system. The presence of *cadA6* in four different novel transposons increases the potential for its dissemination. The *cadA6* resistance determinant has been detected in strains representing four *Listeria* species, including *L. monocytogenes*, isolated from various countries and sources—environmental, food-associated, and clinical samples. We have experimentally confirmed the involvement of the *cadA6* determinant in the increased cadmium tolerance of *Listeria* spp. strains and designed molecular probes to verify the presence of this novel variant in other strains. Previous studies have suggested that some *cadAC* cassettes may additionally influence the phenotypic traits not related to heavy-metal resistance [30,36]. Therefore, the prevalence of various *cadAC* efflux systems and the means of their dissemination should be monitored in *Listeria* spp. strains isolated from different environments.

## Figures and Tables

**Table 1:** Primers used in PCRs to amplify resistance determinants.

Primer	Sequence (5′→3′)	Annealing [Tm, °C ]	Amplified DNA Fragment		Reference
			Targeted Gene (Original Source)	Size [bp]	
**Identification of Resistance Determinants by PCR**					
cadA1-F cadA1-R	CAGAGCACTTTACTGACCATCAATCGTT CTTCTTCATTTAACGTTCCAGCAAAAA	53	*cadA1* (Tn*5422*)	594	[16]
cadA2-F cadA2-R	ACAAGTTAGATCAAAAGAGTCTTTTATT ATCTTCTTCATTTAGTGTTCCTGCAAAT	53	*cadA2* (plM80)	590	[16]
cadA3-F cadA3-R	GCGATGATTGATAATGTCGATTACAAAT TGGTAATTTCTTTAAGTCATCTCCCATT	52	*cadA3* (ICELm1 of EGD-e strain)	468	[16]
cadA4-F cadA4-R	GCATACGTACGAACCAGAAG CAGTGTTTCTGCTTTTGCTCC	51	*cadA4* (genomic island LGI2)	1135	[14]
cadA5-F cadA5-R	GTGATTGTTAATAGGCAGGAAG GTGGTATACCCAACATTG	47	*cadA5* (genomic island LGI2-1)	1178	[52]
cadA6-F cadA6-R	ACTTGTACAAACTGTGCATC AGTAATGTTGCTCCCATATCG	51	*cadA6* (pLIS4, pLIS6)	2027	This study
arsA1-F arsA1-R	CAACTTTGACCCTGTGGAG CTTTCCATTCAATCACTGCG	50	*arsA1* (genomic island LGI2)	1466	[14]
arsA2-F arsA2-R	CAACCAGATCAGTTACCATTAAC TGCTTCTCCAGAGATTTCTTCTG	50	*arsA2* (genomic island LGI2)	1710	[14]
arsATn554F arsATn554R	TAACCAATAAGCCAACACCG CTTCTTTCCACTTCCCGAGC	64	*arsA* (Tn*554*–like transposon)	1219	[15]
FB001 LM2004	CACACTGTAATGTTAACTGG TCCAAGTGCTGAGAATATAC	47	*ctpA* (plasmid-associated)	396	[39]
**Cloning of *cadA6* Genes**					
cadA6aF cadA6aR	GCGACTAGTGCGCCCTCTAATGGAATAAC ATGCGGCCGCTGCTTCTCGCTTCTTTGC	49	*cadA6a* and *cadC6a* cassette (pLIS4, strain Sr12)	2843	This study
cadA6bF cadA6bR	CGCGACTAGTTCTCCAGGTCTCAATTTGTC AAGCGGCCGCCCACAAAAGGTGCTTAGTTC	49	*cadA6b* and *cadC6b* cassette (pLIS6 strain Sr11)	3072	This study

## Supplementary Materials

The following are available online at https://www.mdpi.com/1422-0067/21/22/8713/s1.
