# Supplementary material for: Genetic Carriers and Genomic Distribution of cadA6—A Novel Variant of a Cadmium Resistance Determinant Identified in Listeria spp
Source: Int J Mol Sci. 2020 Nov 18;21(22):8713. doi: 10.3390/ijms21228713 (PMC7698968; doi:10.3390/ijms21228713)
Supplement: Supplementary file 1 [file ijms-21-08713-s001.zip › Figures_S1_S2_S3_S4_S5.pdf]

A

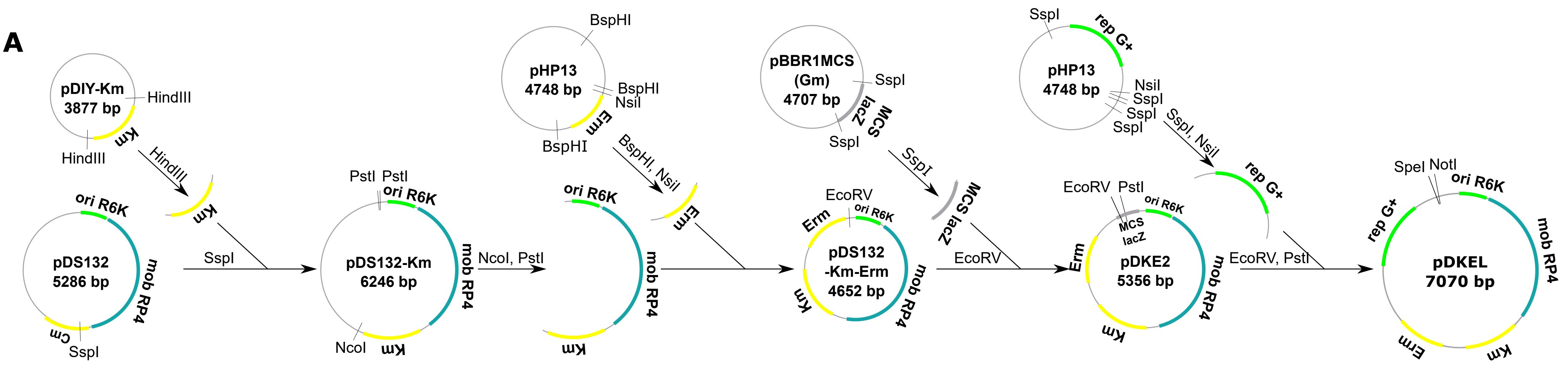

B

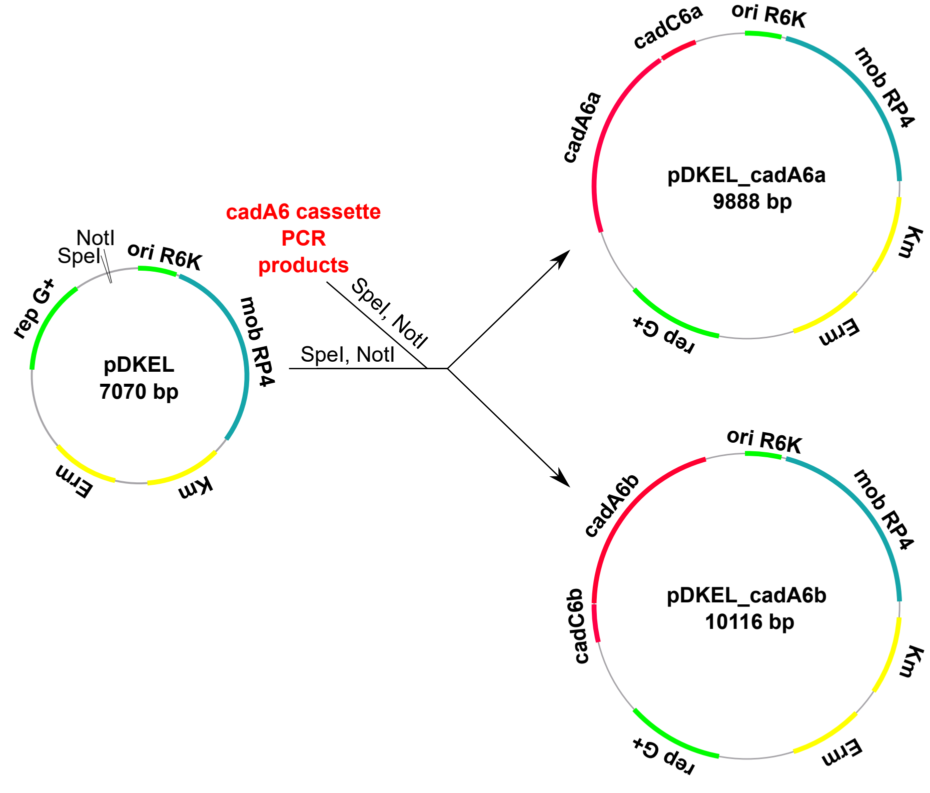

Figure S1. Cloning scheme of plasmids: (A) pDKEL, (B) pDKEL_cadA6a, pDKEL_cadA6b. A simplified structure of plasmids is presented, showing only relevant modules.

- replication module: R6K origin of replication (ori R6K), pTA1060 replication system (rep G+)
- RP4 transfer module (mob RP4)
- resistance determinants: kanamycin (Km), erythromycin (Erm)
- multiple cloning site in lacZ gene (MCS lacZ)
- cadmium resistance cassette: cadC, cadA6

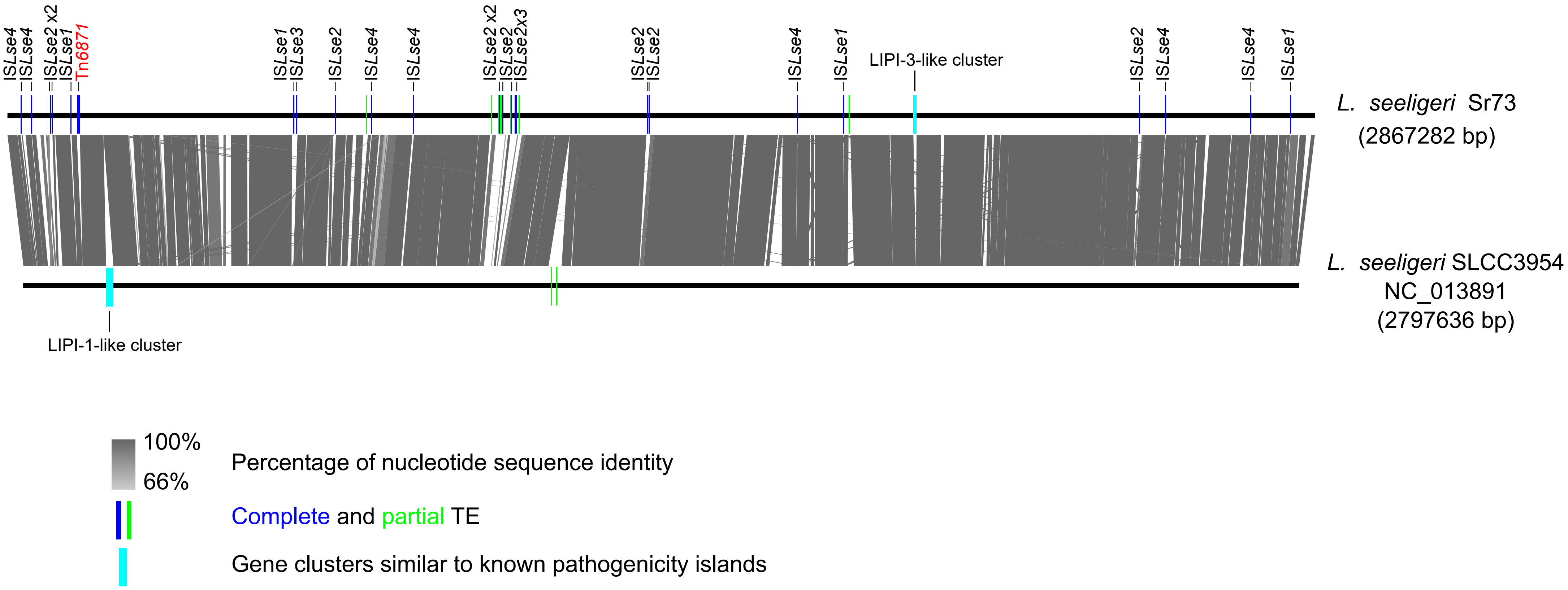

Figure S2. Chromosome synteny analysis of strains *L. seeligeri* Sr73 and *L. seeligeri* SLCC3954 (NC_013891) visualized using EasyFig software.

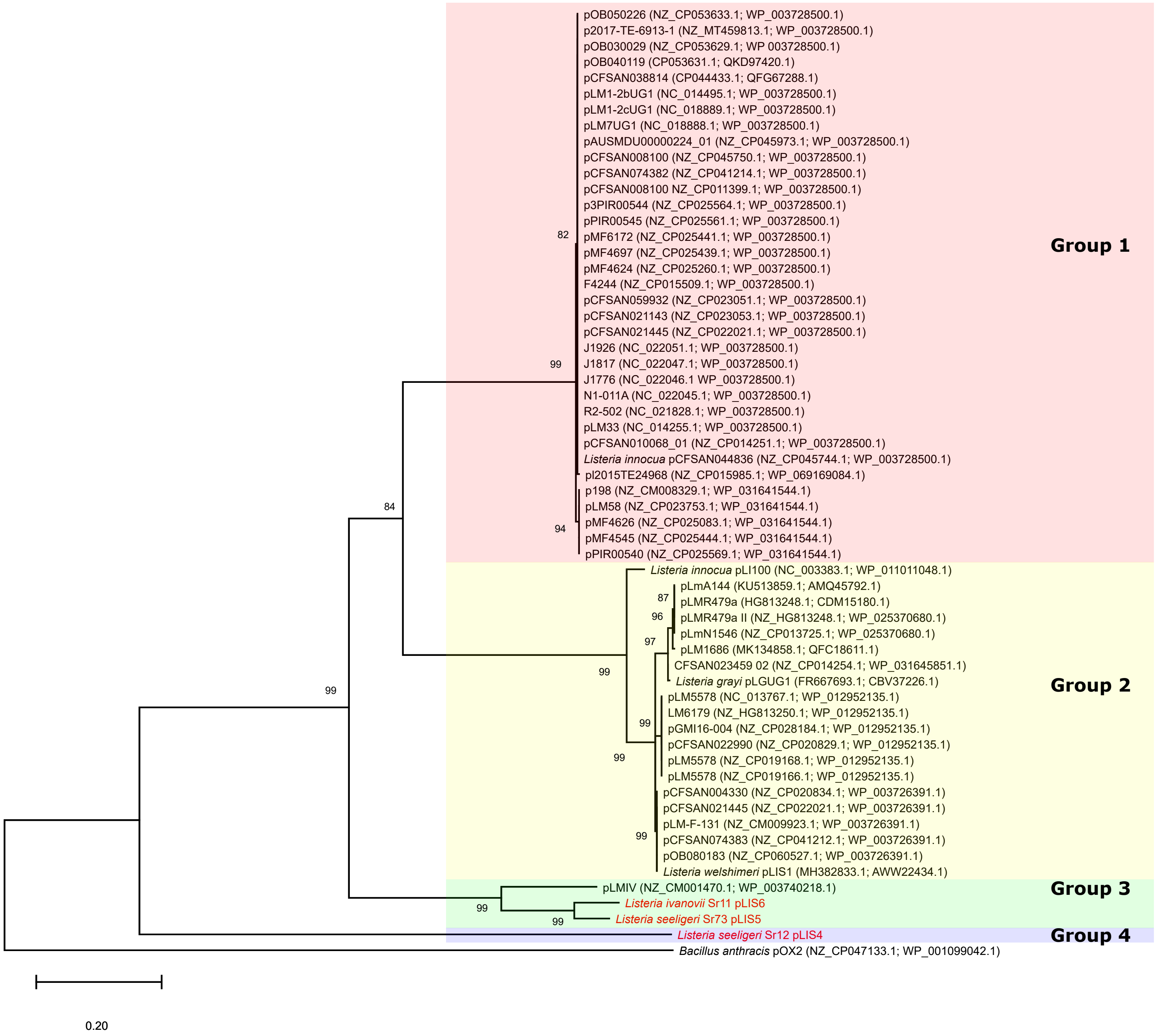

Figure S3. Phylogenetic tree of the RepA proteins of complete *Listeria* spp. plasmid sequences available in GenBank database (as of 30th of August 2020) and pOX2 plasmid of *Bacillus anthracis*. The tree was constructed in MEGAX based on 540 amino acid positions, using the maximum-likelihood algorithm (Le and Gascuel model). The tree is drawn to scale, with branch lengths representing the number of substitutions per site. Statistical support for the internal nodes was determined by 1000 bootstrap replicates. Presented RepA are from *L. monocytogenes* plasmids, unless indicated otherwise. GenBank accession numbers of the nucleotide sequence of the plasmid and the RepA amino acid sequences used for the phylogenetic analysis are given in parentheses. Plasmids sequenced in this study are indicated in red. The RepA of pOX2 from *B. anthracis* was used as an outgroup.

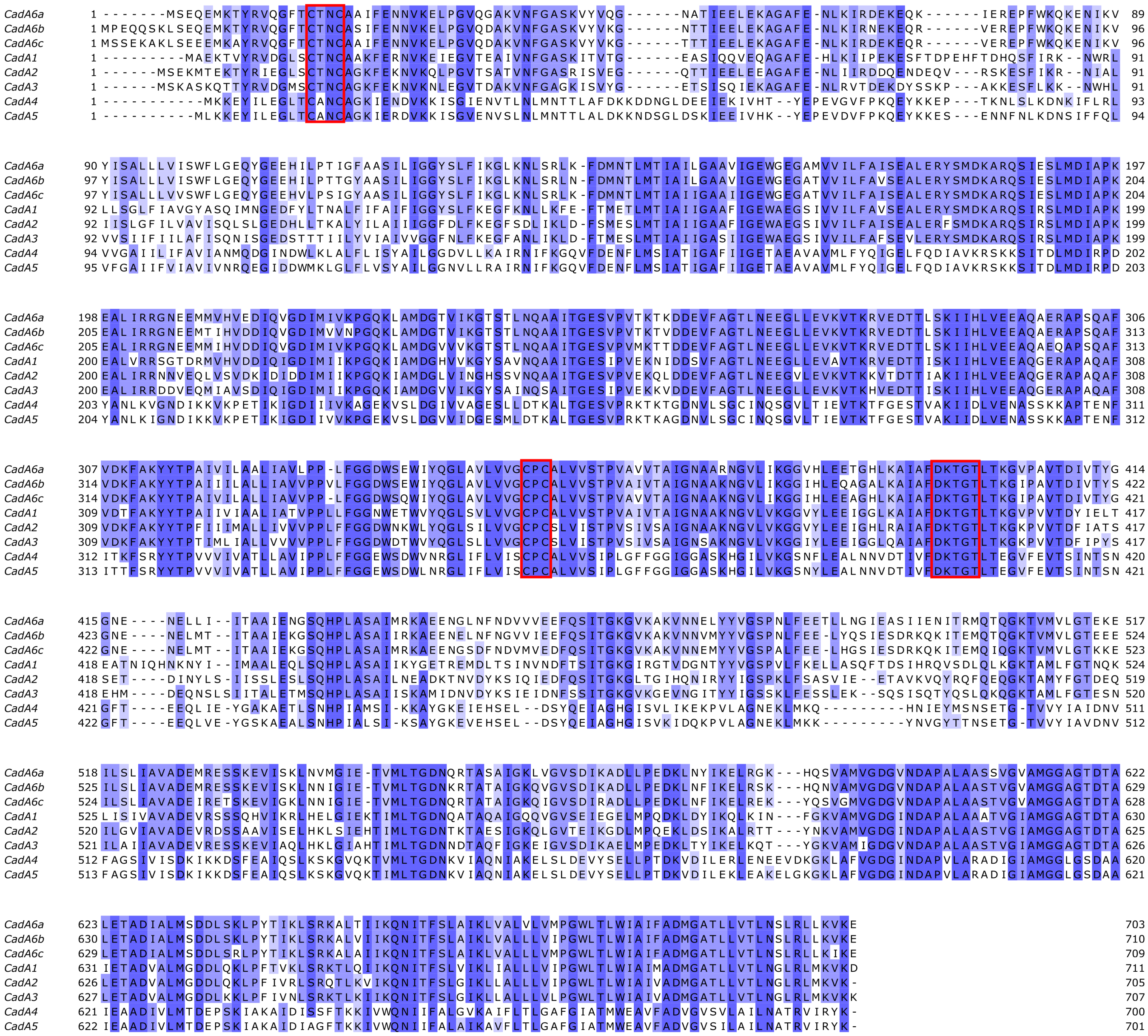

Figure S4. Multiple sequence alignment of different variants of *Listeria* CadA proteins with key motifs marked [78]. CadA1 (GenBank acc. no. WP_003728466.1), CadA2 (WP_003726381), CadA3 (WP_010989665.1), CadA4 (WP_003744464.1), CadA5 (WP_047584121.1), CadA6a (plasmid pLIS4), CadA6b (plasmid pLIS6), CadA6c (WP_031644256.1). Key motifs characteristic of the CadA protein family (CXXC; CPC; DKTGT) are marked in red.

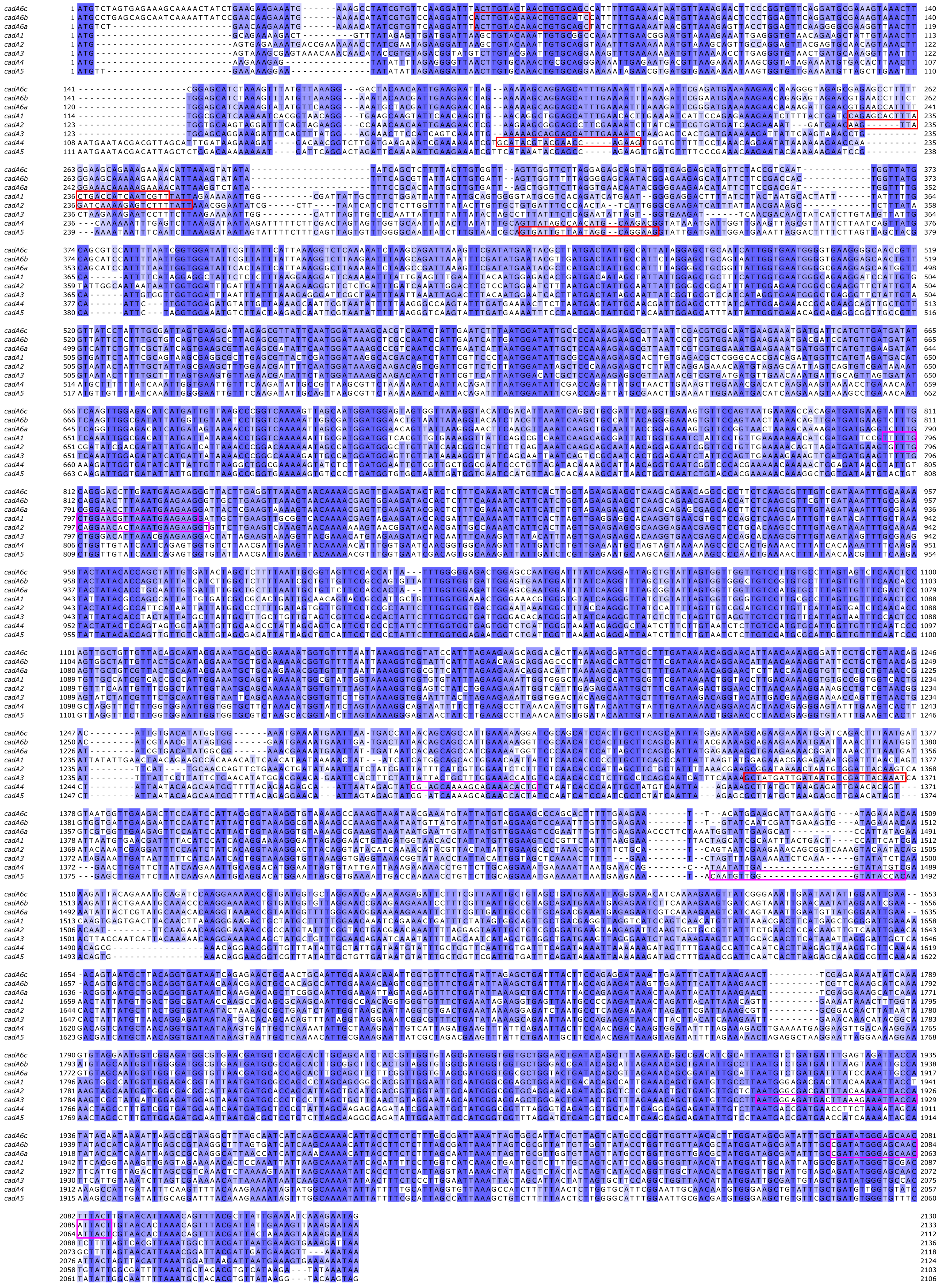

Figure S5. Multiple sequence alignment of different *cadA* gene variants: *cadA1* (GenBank acc. no.L28104.1), *cadA2* (AADR01000058.1), *cadA3* (NC_003210.1), cadA4 (CM001159.1), *cadA5* (NZ_CP007459.1), *cadA6a* (plasmid pLIS4), *cadA6b* (plasmid pLIS6), *cadA6c* (NZ_PQHI01000019.1). Primer pair binding positions for different variants of *cadA* genes are outlined in red (forward) and pink (reverse).
