# Supplementary material for: Genetic Carriers and Genomic Distribution of cadA6—A Novel Variant of a Cadmium Resistance Determinant Identified in Listeria spp
Source: Int J Mol Sci. 2020 Nov 18;21(22):8713. doi: 10.3390/ijms21228713 (PMC7698968; doi:10.3390/ijms21228713)
Supplement: Supplementary file 1 [file ijms-21-08713-s001.zip › TableS1.pdf]

Table S1. Characterization of *Listeria* spp. strains, isolated from water and soil samples, used in this study.

No.	Strain	Source of isolation	**Phenotypic analysis** ■ growth □ lack of growth											Plasmid profile
			**Cadmium chloride**				**Sodium (meta) arsenite**				**Copper(II) sulfate pentahydrate**			
			Concentration [ug/ml]			PCR	concentration [ug/ml]			PCR	concentration [ug/ml]			
			35 (0.19 mM)	75 (0.41 mM)	150 (0.82 mM)		250 (1.92 mM)	500 (3.85 mM)	1000 (7.70 mM)		600 (2.40 mM)	1200 (4.81 mM)	2400 (9.61 mM)	
L. monocytogenes														
1	Sr1	Forest	■	■	■	*cadA1*	□	□	□		■	□	□	p1
2	Sr2	Forest	■	■	■	*cadA1*	□	□	□		■	□	□	p1
3	Sr6	Pine forest, Kampinoski National Park	■	■	■	*cadA1*	□	□	□		■	■	□	p1
4	Sr7	Pine forest, Kampinoski National Park	■	■	■	*cadA1*	□	□	□		■	□	□	p1
5	Sr22	Vegetable garden	□	□	□		□	□	□		■	■	□	
6	Sr35	Garden	■	■	■	*cadA4*	■	■	■	*arsA1*, *arsA2* (LGI2)	■	□	□	
7	Sr44	Pine forest	□	□	□		□	□	□		■	■	□	
8	Sr47	Pine forest	□	□	□		□	□	□		■	■	□	
9	Sr55	City Park „Park Saski”	□	□	□		□	□	□		■	■	□	
10	Sr57	City Park „Park Saski”	□	□	□		□	□	□		■	□	□	
11	Sr91	Forest	□	□	□		□	□	□		■	■	□	
12	Sr106	City	□	□	□		□	□	□		■	■	□	
13	Sr117	City Park	□	□	□		□	□	□		■	□	□	

14	Sr118	City Park	□	□	□		□	□	□		■	■	■	p2
15	Sr119	Wisła River bank	■	■	■	*cadA6*	□	□	□		■	■	□	
16	Sr121	Wisła River bank	□	□	□		□	□	□		■	■	□	p3
17	Sr122	Wisła River bank	□	□	□		□	□	□		■	□	□	
18	Sr125	Wisła River bank	■	■	■	*cadA6*	□	□	□		■	□	□	
19	Sr126	Wisła River bank	■	■	■	*cadA6*	□	□	□		■	■	□	p4
20	Sr130	Forest	■	■	■	*cadA6*	□	□	□		■	□	□	
21	Sr131	Shrubs	■	■	■	*cadA6*	□	□	□		■	■	□	
22	Sr132	Shrubs	■	■	■	*cadA6*	□	□	□		■	□	□	
23	Sr134	City Park „Park Praski”	□	□	□		□	□	□		■	□	□	
24	Sr154	Field (farm)	□	□	□		□	□	□		■	□	□	p5
L. seeligeri														
25	Sr3	City	□	□	□		□	□	□		□	□	□	
26	Sr4	City	□	□	□		□	□	□		□	□	□	
27	Sr5	City	□	□	□		□	□	□		□	□	□	
28	Sr12	Fruit orchard	■	■	□	*cadA6*	■	■	□	*arsA* (Tn*554*-like)	■	■	□	p6 (pLIS4)
29	Sr13	Fruit orchard	■	■	□	*cadA6*	□	□	□		■	■	□	
30	Sr14	Vegetable garden	□	□	□		□	□	□		□	□	□	
31	Sr15	Vegetable garden	□	□	□		□	□	□		■	□	□	
32	Sr17	Vegetable garden	□	□	□		□	□	□		□	□	□	

33	Sr18	Vegetable garden	■	■	□	*cadA6*	□	□	□		■	□	□	
34	Sr23	Vegetable garden	□	□	□		□	□	□		■	■	□	
35	Sr24	Garden	■	■	□	*cadA6*	□	□	□		■	■	□	p6
36	Sr25	Garden	■	■	□	*cadA6*	■	■	□	*arsA* (Tn*554*-like)	■	■	□	
37	Sr26	Garden	■	■	□	*cadA6*	■	■	□	*arsA* (Tn*554*-like)	■	■	□	
38	Sr28	Garden	□	□	□		□	□	□		■	■	□	
39	Sr29	Garden	□	□	□		□	□	□		■	■	□	
40	Sr31	Compost	□	□	□		■	■	□	*arsA* (Tn*554*-like)	■	■	□	
41	Sr32	Compost	■	■	□	*cadA6*	■	■	□	*arsA* (Tn*554*-like)	■	■	□	
42	Sr33	Compost	■	■	□	*cadA6*	■	■	□	*arsA* (Tn*554*-like)	■	■	□	p6
43	Sr34	Compost	■	□	□		■	□	□		■	□	□	
44	Sr36	Garden	□	□	□		□	□	□		■	□	□	
45	Sr40	City Park „Park Saski”	■	■	□	ND[1]	■	■	■	ND	■	■	□	
46	Sr41	Shrubs near fields	□	□	□		■	■	□	ND	■	■	□	
47	Sr43	Pine forest	□	□	□		■	■	□	ND	■	■	□	
48	Sr45	Pine forest	□	□	□		■	■	□	ND	■	■	□	
49	Sr46	Pine forest	■	■	■	*cadA6*	■	□	□		■	■	□	
50	Sr48	Pine forest	■	■	■	*cadA6*	■	■	□	ND	■	■	□	
51	Sr50	City Park „Park Saski”	■	■	■	*cadA1*	□	□	□		■	□	□	p7
52	Sr58	City Park „Park Saski”	■	■	■	*cadA6*	□	□	□		■	□	□	

53	Sr59	City	■	■	■	*cadA1*	■	□	□	*arsA* (Tn*554*-like)	■	■	□	p7
54	Sr60	City	□	□	□		□	□	□		□	□	□	
55	Sr61	City	□	□	□		■	□	□		□	□	□	
56	Sr62	City	□	□	□		□	□	□		■	■	□	
57	Sr64	City	■	■	■	*cadA6*	■	□	□	*arsA* (Tn*554*-like)	■	■	□	
58	Sr65	City	■	■	■	*cadA6*	■	■	□	*arsA* (Tn*554*-like)	□	□	□	
59	Sr66	City	□	□	□		□	□	□		■	■	□	
60	Sr67	City	■	■	■	*cadA6*	■	■	□	*arsA* (Tn*554*-like)	■	■	□	
61	Sr68	City	■	□	□		■	□	□		■	□	□	
62	Sr69	City	■	■	■	*cadA6*	■	■	□	*arsA* (Tn*554*-like)	■	■	□	
63	Sr70	City	■	■	■	*cadA6*	■	□	□	*arsA* (Tn*554*-like)	■	■	□	
64	Sr71	City	■	■	■	*cadA6*	■	■	□	*arsA* (Tn*554*-like)	■	■	□	p6
65	Sr72	City	■	■	■	*cadA6*	■	□	□	*arsA* (Tn*554*-like)	■	■	□	
66	Sr73	Dry pine forest	■	■	■	*cadA6*	■	■	□	*arsA* (Tn*554*-like)	■	□	□	p8 (pLIS5)
67	Sr74	Dry pine forest	■	■	■	*cadA6*	■	■	□	*arsA* (Tn*554*-like)	■	□	□	p8
68	Sr75	Dry pine forest	■	■	■	*cadA6*	■	■	□	*arsA* (Tn*554*-like)	■	■	□	
69	Sr77	Dry pine forest	■	■	■	*cadA1*	■	□	□		■	□	□	p9
70	Sr78	Dry pine forest	■	■	■	*cadA1*	□	□	□		■	■	□	p7
71	Sr80	Dry pine forest	□	□	□		■	□	□		□	□	□	
72	Sr81	Dry pine forest	■	■	■	*cadA6*	■	■	□	*arsA* (Tn*554*-like)	■	■	□	

73	Sr85	City Park „Pola Mokotowskie”	□	□	□		■	■	□	ND	■	■	□	
74	Sr86	Forest, near river bank	□	□	□		■	□	□		■	■	□	
75	Sr87	Forest, near river bank	□	□	□		■	□	□		■	■	□	
76	Sr88	Forest, near river bank	□	□	□		□	□	□		■	■	□	
77	Sr89	Forest	□	□	□		□	□	□		■	■	□	
78	Sr93	Forest	□	□	□		□	□	□		■	■	□	
79	Sr94	Forest	□	□	□		■	□	□		■	■	□	
80	Sr95	Forest	□	□	□		□	□	□		■	□	□	
81	Sr97	Forest	□	□	□		□	□	□		■	□	□	
82	Sr99	City Park „Park Saski”	□	□	□		■	□	□		□	□	□	
83	Sr100	City Park „Park Saski”	□	□	□		■	■	□	*arsA* (Tn*554*-like)	■	■	□	
84	Sr120	Wisła River bank	■	■	□	ND	■	■	□	ND	■	□	□	
85	Sr123	Wisła River bank	□	□	□		□	□	□		■	□	□	
86	Sr124	Wisła River bank	□	□	□		□	□	□		■	□	□	
87	Sr127	Wisła River bank	□	□	□		■	□	□		□	□	□	p10
88	Sr128	Wisła River bank	■	■	■	*cadA6*	■	■	□	*arsA* (Tn*554*-like)	■	□	□	
89	Sr129	Wisła River bank	□	□	□		■	■	□	ND	■	□	□	
90	Sr140	Field (farm)	□	□	□		■	□	□		■	□	□	
91	Sr141	Field (farm)	□	□	□		□	□	□		■	■	□	
92	FSL S4-0003	USA, pristine vegetation	□	□	□		□	□	□		■	■	□	

93	FSL S4-0057	USA, pristine soil	■	■	□	*cadA1*	□	□	□		■	■	□	p11
94	FSL S4-0073	USA, pristine soil	□	□	□		■	■	□	ND	■	□	□	
L. innocua														
95	Sr9	Swamps, Kampinoski National Park	□	□	□		□	□	□		■	■	□	
96	Sr10	Swamps, Kampinoski National Park	□	□	□		□	□	□		■	□	□	
97	Sr49	City Park „Park Saski”	■	■	□	*cadA6*	□	□	□		■	■	□	
98	Sr51	City Park „Pola Mokotowskie”	□	□	□		□	□	□		■	■	□	
99	Sr52	City Park „Park Saski”	□	□	□		□	□	□		■	□	□	
100	Sr53	City Park „Park Saski”	■	■	□	*cadA1*	■	□	□		■	■	□	
101	Sr56	City Park „Park Saski”	□	□	□		□	□	□		■	□	□	
102	Sr90	Forest	□	□	□		□	□	□		■	■	□	
103	Sr92	Forest	□	□	□		□	□	□		■	■	□	
104	Sr96	City Park „Pola Mokotowskie”	□	□	□		□	□	□		■	■	□	
105	Sr98	Swamps	□	□	□		□	□	□		■	■	□	
106	Sr101	Vegetable garden	□	□	□		□	□	□		■	■	□	
107	Sr102	City Park	□	□	□		□	□	□		■	■	□	p12
108	Sr103	City Park	■	■	■	*cadA6*	□	□	□		■	□	□	
109	Sr104	City Park	□	□	□		□	□	□		■	□	□	
110	Sr105	City Park	■	■	■	ND	□	□	□		■	■	□	p13
111	Sr107	City	□	□	□		□	□	□		■	■	□	

112	Sr108	City	■	■	■	*cadA1*	□	□	□		□	□	□	p14
113	Sr109	City	■	■	■	*cadA1*	□	□	□		□	□	□	p14
114	Sr110	City	□	□	□		□	□	□		■	□	□	
115	Sr111	City	□	□	□		□	□	□		■	□	□	
116	Sr112	City	■	■	■	*cadA6*	□	□	□		■	■	□	
117	Sr113	City	□	□	□		□	□	□		■	■	□	
118	Sr114	City	□	□	□		□	□	□		■	■	□	p15
119	Sr115	City	□	□	□		□	□	□		■	■	□	p15
120	Sr116	City	□	□	□		□	□	□		■	■	□	
121	Sr136	City Park „Park Praski”	□	□	□		□	□	□		■	■	□	
122	Sr137	City Park „Park Praski”	□	□	□		□	□	□		■	■	□	
123	FSL H4-0088	USA, water	□	□	□		□	□	□		■	□	□	
124	Sr155	Field (farm)	□	□	□		□	□	□		■	□	□	
L. ivanovii														
125	Sr8	Swamps, Kampinoski National Park	□	□	□		□	□	□		■	□	□	
126	Sr11	Fruit orchard	■	■	□	*cadA6*, *cadA1*	■	□	□	*arsA* (Tn*554*-like)	■	■	□	p16 (pLIS6)
127	Sr16	Vegetable garden	■	■	□	*cadA6*	■	□	□	*arsA* (Tn*554*-like)	□	□	□	p16
128	Sr19	Vegetable garden	■	■	□	*cadA1*	■	□	□	*arsA* (Tn*554*-like)	□	□	□	p17
129	Sr20	Vegetable garden	■	□	□		■	□	□	*arsA* (Tn*554*-like)	■	■	□	
130	Sr21	Vegetable garden	■	□	□		■	□	□	*arsA* (Tn*554*-like)	■	□	□	

131	Sr27	Garden	□	□	□		□	□	□		■	□	□	
132	Sr30	Manure	□	□	□		■	□	□	*arsA* (Tn*554*-like)	■	□	□	
133	Sr37	Forest	□	□	□		□	□	□		□	□	□	
134	Sr39	Shrubs near fields	□	□	□		□	□	□		■	□	□	p18
135	Sr42	Forest	■	□	□		■	□	□	*arsA* (Tn*554*-like)	■	■	□	
136	Sr63	City Park	■	■	□	*cadA1*	■	□	□	*arsA* (Tn*554*-like)	□	□	□	
137	Sr76	Forest	■	■	□	*cadA6*	■	□	□	*arsA* (Tn*554*-like)	□	□	□	
138	Sr79	Dry pine forest	■	■	□	*cadA1*	■	□	□	*arsA* (Tn*554*-like)	■	■	□	
139	Sr82	Dry pine forest	□	□	□		□	□	□		□	□	□	
140	Sr139	Field (farm)	□	□	□		□	□	□		□	□	□	p19
L. welshimeri														
141	Sr38	City Park „Park Saski”	□	□	□		□	□	□		■	■	□	
142	Sr54	City Park „Park Saski”	□	□	□		□	□	□		■	■	□	
143	Sr83	City Park „Pola Mokotowskie”	□	□	□		□	□	□		■	□	□	
144	Sr84	City Park „Pola Mokotowskie”	□	□	□		□	□	□		■	■	□	
145	Sr133	City Park „Park Praski”	□	□	□		□	□	□		■	□	□	
146	Sr135	City Park „Park Praski”	□	□	□		□	□	□		■	□	□	
147	Sr138	City Park „Park Praski”	□	□	□		□	□	□		■	□	□	
148	FSL H4-0277	USA, water	□	□	□		□	□	□		□	□	□	
149	FSL S4-0070	USA, pristine soil	□	□	□		□	□	□		■	□	□	
150	FSL S4-	USA, pristine water	□	□	□		□	□	□		■	□	□	

	0101													
151	FSL S4-0105	USA,pristine field	□	□	□		□	□	□		■	□	□	
L. marthii														
152	FSL S4-0120	USA, pristine forest soil	□	□	□		□	□	□		■	□	□	
153	FSL S4-0696	USA, pristine standing water	□	□	□		□	□	□		■	□	□	
154	FSL S4-0710	USA, pristine flowing water	□	□	□		□	□	□		■	□	□	
155	FSL S4-0965	USA, pristine flowing water	□	□	□		□	□	□		■	□	□	

[1] ND – not determined resistance determinant
